# Muscle function decline and mitochondria changes in middle age precede sarcopenia in mice

**DOI:** 10.18632/aging.101358

**Published:** 2018-01-04

**Authors:** Andrea del Campo, Ignacio Contreras-Hernández, Mauricio Castro-Sepúlveda, Cristian A. Campos, Reinaldo Figueroa, María Florencia Tevy, Verónica Eisner, Mariana Casas, Enrique Jaimovich

**Affiliations:** 1 Center for Exercise, Metabolism and Cancer, ICBM, Faculty of Medicine, Universidad de Chile, Santiago, Chile; 2 Department of Cellular and Molecular Biology, School of Biological Sciences, Pontificia Universidad Católica de Chile, Santiago, Chile; 3 Center for Genomics and Bioinformatics, Universidad Mayor de Chile, Santiago, Chile; 4 Physiology Program, ICBM, Faculty of Medicine, Universidad de Chile, Santiago, Chile; 5 Facultad de Medicina, Pontificia Universidad Católica de Chile, Santiago, Chile

**Keywords:** skeletal muscle fibers, sarcopenia, mitochondrial dynamics, SR-mitochondria coupling

## Abstract

Sarcopenia is the degenerative loss of muscle mass and strength with aging. Although a role of mitochondrial metabolism in muscle function and in the development of many diseases has been described, the role of mitochondrial topology and dynamics in the process of muscle aging is not fully understood. This work shows a time line of changes in both mitochondrial distribution and skeletal muscle function during mice lifespan. We isolated muscle fibers from *flexor digitorum brevis* of mice of different ages. A fusion-like phenotype of mitochondria, together with a change in orientation perpendicular to the fiber axis was evident in the Adult group compared to Juvenile and Older groups. Moreover, an increase in the contact area between sarcoplasmic reticulum and mitochondria was evident in the same group. Together with the morphological changes, mitochondrial Ca^2+^ resting levels were reduced at age 10-14 months and significantly increased in the Older group. This was consistent with a reduced number of mitochondria-to-jSR pairs in the Older group compared to the Juvenile. Our results support the idea of several age-dependent changes in mitochondria that are accentuated in midlife prior to a complete sarcopenic phenotype.

## Introduction

One of the most pronounced changes in the elderly is the loss of mobility and physical capacity, degrading the quality of life. These changes occur due to a progressive loss of skeletal muscle mass and function, a process known as sarcopenia [1]. After the age of 25, muscle mass declines 3% to 10% per decade [2]; and reaches a speed of 1% decline per year at older ages [3]. This disability is independent of ethnicity, age, morbidity, obesity, income, or health behaviors, and is a major public health problem worldwide [4]. Moreover, alterations in skeletal muscle may possibly lead to other diseases that appear during aging such as a decrease in metabolic rate, increased insulin resistance and bone loss [5].

Recent evidence has suggested that sarcopenia can occur not only because of aging but also as an outcome of other aging-associated pathologies. Despite these new considerations, the definitions of sarcopenia are now focused on establishing loss of muscle function more than muscle mass loss as a potential predictive model of frailty in old people [6]. Some authors have been particularly interested in late-life interventions to prevent sarcopenia symptoms or improve sarcopenia markers and outcomes, on this regard it has been described that calorie restriction and some pharmacological interventions may improve physical capacity only when a time frame for the intervention is identified [7].

Sarcopenia can be caused by decreased contractile elements [8] by reducing the total number of muscle fibers, by decreasing the size of fast twitch type II muscle fibers or by a loss of motor units [9]. However, the mechanisms underneath these changes have been only partially understood. Thus, the cellular and molecular mechanisms that underlie the functional loss in aging skeletal muscle need to be studied in detail.

Some of the changes at the cellular level, seen in aged muscle cells include accumulation of intra or extracellular lipids; misfolding of structural and contractile proteins, and mitochondrial dysfunction [10]. Current evidence suggests that the decreases in mitochondrial respiratory enzymes, especially complex IV [11]. the decrease in the mitochondrial content and also increases in Carbonic anhydrases associated with mitochondria [12] appear to be key factors in the process of muscle aging, as demonstrated by a reduction in both mtDNA copies, and tricarboxylic acid cycle enzymes activity [2] [13]. One of the main theories of cellular aging establishes that there is a strong positive correlation between age and oxidative damage [14]. On this regard, recent studies demonstrated that oxidative stress contribute to mitochondrial dysfunction but is not related with muscle fiber atrophy, separating oxidative stress from muscle mass loss in sarcopenia [15]. Other studies have documented that mitochondrial dynamics events can respond to different stimuli promoting or decreasing mitochondrial bioenergetics and metabolism [16]. Nevertheless, the association between mitochondrial morphology and aging remains to be unveiled.

Nowadays, there is significant evidence supporting a close relationship between mitochondrial function and morphology in skeletal muscle [17] [18]. A decrease in muscle mitochondrial volume, density and function has also been found with age, but other studies support the fact that maintenance or even a tendency towards increased mitochondrial density occur during the aging process; moreover differential mitochondrial and glycolytic enzymes changes have been reported in different fiber types [19]. In the context of studies examining mitochondrial morphology with aging, Leduc-Gaudet et al. reported highly fused mitochondrial structure in aged mouse muscle [20].

Altogether the existing evidence suggest that there is a relationship between mitochondrial morphology and aging which can even depend on muscle fiber type and that has not been completely elucidated, being still a matter of controversy [21].

In fully differentiated skeletal muscle fibers, there is evidence that mitochondrial membranes interact *in vivo* [21]. In addition, fusion events occur and become less frequent than in undifferentiated myoblasts and generally result in a more specialized and durable matrix domains complementation [22]. These changes can be attributed, at least in part, to the highly organized architecture of the muscle fiber, which offers few possibilities for movement of the mitochondria to join and separate from other individual mitochondria [23,24]. Mitochondrial morphology in mature myofibers consists of two divergent but interacting populations of mitochondria, for which size and morphological parameters have been described. Sub-sarcolemmal (SSM) and intermyofibrillar (IMF) are proposed to form an interconnected network of both types of mitochondria in adult muscle fibers [25].

Boncompagni et al. reported that FDB muscles from 0.5 to 1 month old mice have more elongated and less organized mitochondria than the muscle fibers of adult mice. In the adult, the majority of the globular IMF mitochondria are positioned in the I band, in apposition to the terminal cisternae of the sarcoplasmic reticulum and the T tubule where the Calcium release units (triads) are formed [26]. Recently, a great interest has been generated by the link between mitochondrial and the endoplasmic reticulum [27]. After establishing the existence of communication between organelles, several questions have raised that aim to find the role of this communication in the basic functioning of the cell.

We previously showed that the relationship between the morphology of the mitochondrial network and its Ca^2+^ buffer capacity in muscle cell lines; a fused phenotype correlates with a higher Ca^2+^ uptake after an insulin stimulus. On the other hand, small mitochondria show reduced Ca^2+^ uptake after stimulation [28]. Moreover it has been proposed that the impairment and miss-position of DHPR and RyR in adult skeletal muscle fibers can be a possible cause of strength decline in aging muscle, where alterations in the ultrastructure and spatial reorganization of the Ca^2+^ release units can be found [29].

In this work, we demonstrate the existence of changes in mitochondrial distribution, topology and morphology in stages prior to old age, these changes appear to be implicated in the mechanism underlying the changes in muscular function that occur gradually during the aging process, and some of these changes correlate well with the appearance of the first symptoms of sarcopenia.

## RESULTS

### Muscle function decline precedes appearance of physical characteristics of sarcopenia

Mice were divided into four groups by age. Groups were defined as juvenile (J) 2-3 months, young (Y) 6-9 months, Adult (A) 10-14 months and Older (O) 16-20 months. The definition of these groups is based in prior research that demonstrates that the complete differentiation of the ultrastructure of the muscle fibers is reached at 4 months [26]. On this regard, we studied a Juvenile group and a Young group to establish the first changes and both an Adult group and an Older group to describe the changes that occur after the young stages of mice lifespan before the dramatic loss of muscle function reported in an oldest stage.

**Figure 1:**
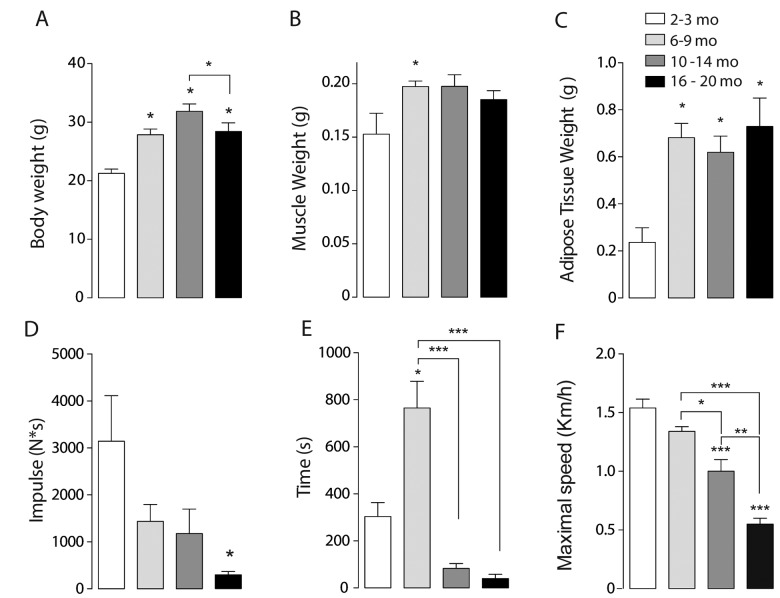
**Sarcopenia symptoms begin at early stages of life in mice.** Each bar represents mean ± SD of 6-8 mice per group. *p<0.05. **p<0.01 ***p<0.001. (**A**) Body Weight reached a maximum peak in the 10-14 months old group. (**B**) Muscle Weight increased significantly in the 6-9 months old group. (**C**) Epididymal Adipose Tissue Weight increased significantly in the three adult groups when compared with juvenile mice. (**D**) Resistance was determined by the time mice kept running at an 80% of maximal speed reached by each animal and was significantly decreased in the older groups (**E**) Impulse (N*s) was determined by Grip assay and is significantly decreased in the Older group (**F**) Maximal Speed significantly decreased in the older groups.

To establish the presence of sarcopenia symptoms, physical parameters as body weight, muscle weight and adipose tissue weight were measured. Body weight significantly increased from 6-9 months on, compared to the juvenile group. Moreover, the maximum body weight was reached in the Adult group and then significantly decreased in the Older group (Fig. 1A). To separate the effect of muscle and adipose tissue over the whole-body weight, we measured the weight of the *gastrocnemius-soleus* complex in the four groups. Muscle weight increased significantly in the Young group compared to the juvenile group. This significant increase is lost in the older groups, supporting the idea that muscle mass begins to decline in middle-aged mice (10-14 months) (Fig. 1B). Epididymal adipose tissue also increased in the 6-9 months group and remains high in the older groups (Fig. 1C). To further investigate the characteristics of sarcopenia we also determined cross-sectional area and collagen infiltration in *gastrocnemius/soleus* muscles of mice in the different groups. Our results show that collagen infiltration is significantly higher in mice of the 16-20 month old group, whereas cross-sectional area significantly decreased in the 10-14 month old group. These results hold the hypothesis that separates muscle function decline from muscle mass loss in the course of sarcopenia symptoms appearance (Supplementary Fig. 1).

To establish loss of muscle function in the older groups when compared to juvenile mice, an inverted grip assay was executed to each group of mice; our results show that impulse (N*s), given by the time mice kept hanging normalized by their own weight, was significantly decreased in the older group when compared to the younger groups (Fig. 1D). To investigate the changes of resistance exercise performance during the aging process we measured maximal velocity and the maximum running time at an 80% of the maximum speed reached by each mouse in an acute treadmill exercise. Our results show that there is a significant decline among the groups in maximal speed, measured by an incremental test, showing significant differences in the 10-14 months group and the 16-20 months old group as opposed to Juvenile and Young mice (Fig. 1F). Thus, a decrease in the maximum speed is consistent with the signs of sarcopenia and therefore consistent with our hypothesis that these changes occur in relatively early stages of life. Moreover, resistance showed a significant increase in the Young group followed by a significant decrease in both 10-14 mo as in 16-20 mo groups (Fig. 1E) when compared to the 6-9 mo group.

Altogether, these physical characteristics show that there are changes in muscle function that occur previous to an old stage of lifespan in mice, and that can be presented as mild sarcopenia symptoms (muscle and weight loss) as characterized in human.

After we established the functional changes in muscle, we studied the changes in mitochondrial morphology during the aging process comparing the same groups of mice.

### Skeletal muscle fibers present age-dependent differences in mitochondrial morphology

To monitor the mitochondrial network, skeletal muscle fibers from the *Flexor Digitorium Brevis* (FDB) were electroporated *in vivo* with Mito-ds-Red plasmid. After one week, isolated FDB fibers were seeded and *in vitro* monitored by live cell confocal microscopy. We detected age-dependent changes in mitochondria morphology as given by number of mitochondria and volume (in pixel^3^ = voxels) after 3D reconstruction.

**Figure 2:**
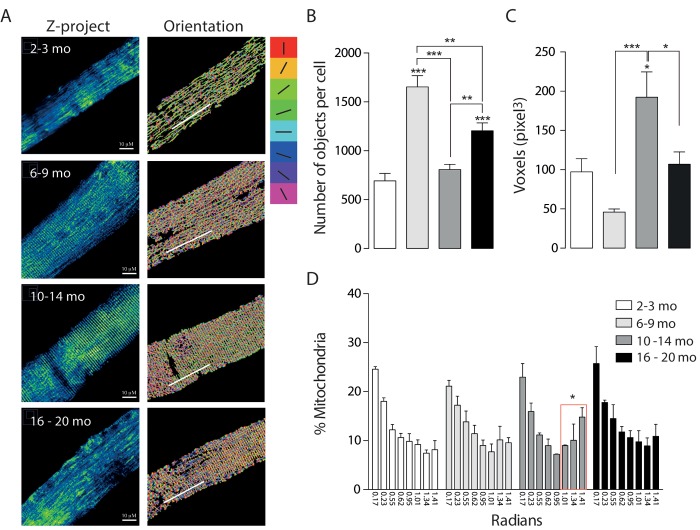
**Mitochondrial morphology and orientation are different in the different age groups.** Each bar represents the mean ± SD of 4-5 mice. *p<0.05. **p<0.01 ***p<0.001 (**A**) Representative Confocal images of isolated FDB muscle fibers electroporated with Mito-DsRed for each age group are shown in pseudo-color. Right panel shows the different orientations of mitochondria given in the analysis in different colors (**B**) Number of mitochondria per cell is significantly increased in the Young (6 - 9 mo) group. (**C**) Volume of mitochondria is significantly increased in the adult group when compared with young and old. (**D**) Histogram representing the orientation of mitochondria shows an important shift from longitudinal towards a transverse orientation in the middle age group.

Juvenile mice from the 2-3 months group were considered as control. Our results show that the Young group (6-9 months old) presented increased number of individual mitochondria together with a tendency to decrease the volume of each mitochondrion in comparison with the Juvenile group (Fig. 2). On the contrary, skeletal muscle fibers from the 10-14 months adult group, showed a significant decrease in the number of objects compared with the Young group, accompanied by a significant increase in the volume; these findings suggest a fused-like phenotype of the mitochondrial network that occurs after the appearance of a first fragmented phenotype seen in the former group of mice at a younger age (Fig. 2). Additionally, the older group presents different morphology (Fig. 2D) characterized by an increase in the number of objects per fiber when compared with either the juvenile group or the adult (10-14) group; moreover, there was a significant decrease in mitochondrial volume in the older group (Fig. 2C). Altogether, these results suggest that the mitochondria size and shape varies with age changing in a non-linear way from fragmented to fused-like phenotype depending on the stage of life of mice.

We also analyzed the re-distribution of the mitochondrial network in isolated muscle fibers from mice of the different groups. To record and quantify the changes observed on mitochondrial distribution in the 3D reconstructions, a semi-automatic segmentation was applied to the muscle cell images. Images showed in the right panel Fig. 2A represent each of the different orientations in Radians in in which a different color was assigned to each orientation. Our results show that in the first two groups about 50% of the mitochondria presented a longitudinal pattern along the fiber axis (Fig. 2D). This longitudinal pattern does not differ significantly between the different groups while the proportion (%) of mitochondria located perpendicular to the major fiber axis displayed a significant increase in the Adult group (31.6+- 13.13%); this redistribution of mitochondria occurs at the same age the mitochondrial network presented a fused-like phenotype (Fig. 2B, C); moreover this redistribution is subsequently lost in the Older group (24.62+-13.99%), where we also recorded the loss of the fused-like phenotype in the mitochondrial network.

**Figure 3:**
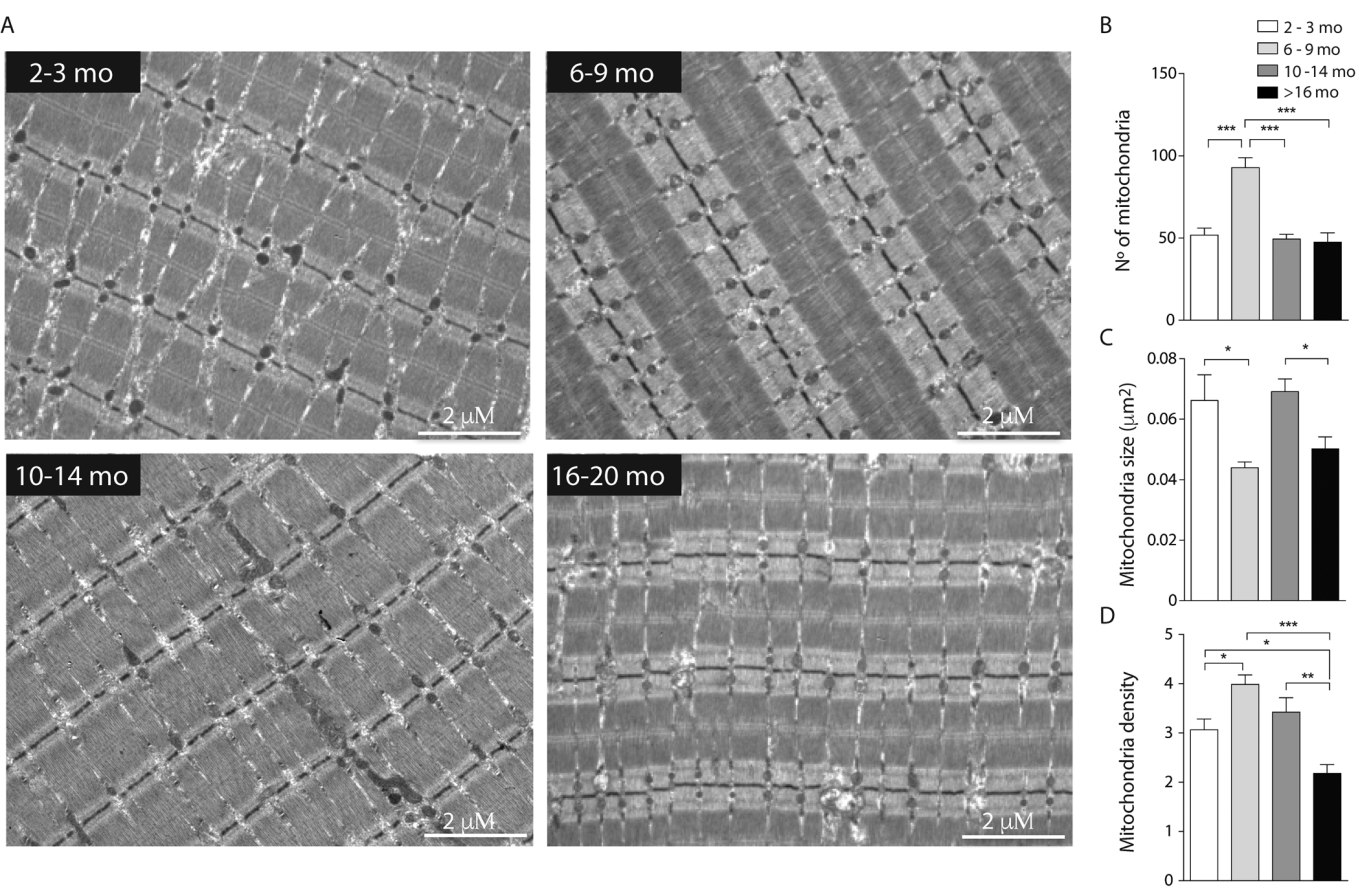
**Decreased mitochondrial size and density in aged mice.** Each bar represents the mean ± SD. *p<0.05. **p<0.01 ***p<0.001 (**A**) Representative images of electron microscopy from FDB longitudinal slices from mice from the different groups (**B**) number of mitochondria per image area (98 μm^2^) is significantly increased in the adult group. (**C**) Mitochondria size is significantly diminished in the young and old groups (**D**) mitochondrial density, given by area of mitochondria/ area of tissue significantly decreased in old stages of life in mice.

To further study the changes in mitochondrial spatial distribution and the status of development of the muscle fiber, electron microscopy for longitudinal slices of FDB muscle was performed (Fig. 3). Our results show that the number of mitochondria significantly decreased in the adult group as the area of mitochondria increased, supporting what we observed in isolated fibers by confocal microscopy (Fig. 3B). Moreover, the area of the mitochondria observed at 6-9 months was significantly decreased suggesting a fragmented phenotype (Fig. 3C). Together with these findings, the mitochondrial density, considered as the area of mitochondria per area of muscle ﬁber, was significantly increased in the Young group when compared to the Juvenile; this is maintained in the Adult group and then significantly decreases in the older group (Fig. 3D). To verify our results, we observed SSM in different ages and quantified number of mitochondria and area by TEM. The number of mitochondria was significantly increased in the subsarcolemma region in the 6-9 months old group, comparable with our observations in the IMF (Supplementary Fig. 2); the changes in this parameter were consistent in both subpopulations of mitochondria in the four age groups. The size of both subsarcolemmal and intermiofibrillar subpopulations of mitochondria mostly correlate with what we found in confocal microscopy, in both we can find an increase in the number of mitochondria for the 6-9 mo group and a subsequent decrease in the older groups, while mitochondria size variations differ in the SS subpopulation, but despite the differences in apparent size, both subpopulations show changes during the aging process that can be clearly differentiated in the four stages.

**Figure 4:**
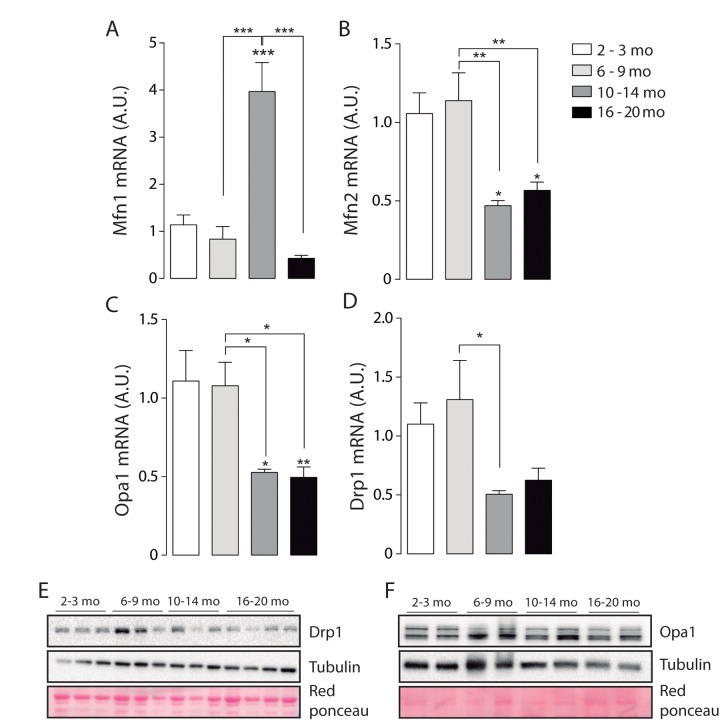
**mRNA levels of proteins involved in mitochondrial dynamics vary during the aging process.** *p<0.05. **p<0.01 ***p<0.001 (**A**) mRNA levels of Mfn1 significantly increased in the adult group. (**B**) mRNA levels of Mfn2 are significantly decreased in the older groups (10-14 mo and 16-20 mo) (**C**) mRNA levels of Opa1 are significantly decreased in the older groups (10-14 mo and 16-20 mo) (**D**) mRNA levels of Drp1 are significantly decreased in the 10-14 mo group. (**E**) mRNA levels of Fis1 are significantly decreased in the 10-14 mo group. (**F**) and (**G**) Representative Western blots of Drp1 and Opa1 respectively.

To further understand the data obtained by microscopy, we quantified mRNA levels of the mitochondrial proteins involved in fusion/ fission processes. Our results show that mRNA for Mfn1 significantly increased in the adult group compared with the Juvenile group (Fig. 4A). Furthermore, Mfn1 mRNA levels significantly decreased in the older group when compared to the Adult. mRNA levels of Opa1 and Mfn2 significantly decrease in both the Adult and Older group (Fig. 4 B-C). Moreover, the fission-related gene *Drp1* was significantly decreased in the adult group (Fig. 4 D). No differences were found in the protein levels of Opa1 (Fig. 4E) or Mfn2 (Supplementary Fig. 3), while Drp1 protein levels were significantly increased in the 6-9 months group when compared to the other age groups (Fig. 4F). To separate the effects of biogenesis in our model we determined the levels of COX IV and TOM 20, two non-related mitochondrial proteins, in skeletal muscle of mice in the different groups, there were no changes when mitochondrial dynamics associated proteins were normalized by COX IV or TOM20 (Supplementary Fig. 3A and B). Moreover, the level of both proteins did not significantly differ in soleus muscle (Supplementary Fig. 3C).

These results show that mitochondrial fusion-fission mRNA and protein levels differentially vary during the aging process, reaching significantly lower levels of mitochondrial dynamics proteins in older animals.

Altogether the ensemble of results shows that the mitochondrial network changes alternatively towards fused or fragmented-like states during mice lifespan; these changes are more prominent in the 10-14 month group and appear to correlate with muscle functional changes in these animals.

### jSR-Mitochondria couplings are impaired in aging mice

Junctional SR-to-mitochondria coupling has been a focus of interest during the last decade. The importance of this communication relies in Ca^2+^ transference from SR to mitochondria, to address intracellular Ca^2+^ homeostasis and support mitochondrial function [30].

**Figure 5:**
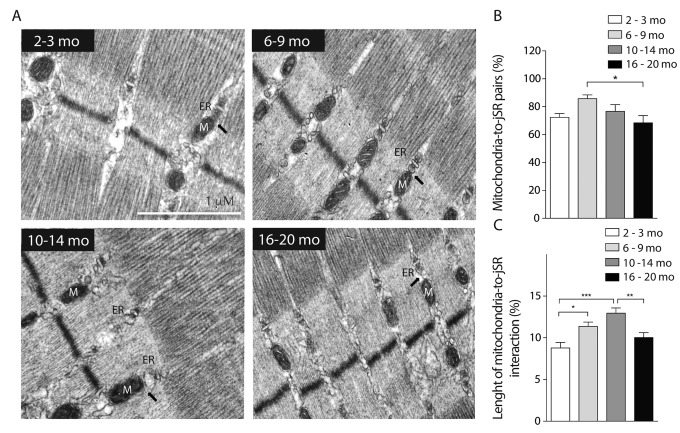
**SR-mitochondria coupling are decreased in aged mice.** (**A**) Representative images of electron microscopy 20.000X from FDB longitudinal slices of mice from the different groups. (**B**) Analysis of ER- mito contacs in percentage shows a significant decrease of these structures in the older group when compared to the 6-9 months old group. (**C**) Length of the SR- mito interface is significantly increased in the adult group and significantly decreased in the old group.

Boncompagni et al. described changes in the position of mitochondria towards the triad during development and finally at 4 months old, mitochondria are fully tethered to the Calcium Release Units [26]. Our results show that during the life span jSR-to-mitochondria pairs increase in the Young group when compared to Juvenile, as expected. Then SR-Mitochondria pairs start to decline until a significant decrease is evident in the 16-20 months old group (Fig. 5B). Moreover, our analysis shows that the length of the SR-mitochondria interface normalized by mitochondrial perimeter is significantly increased in the Young group (6-9 Mo) and further increased in the Adult group (10-14 Mo) to finally significantly decrease in the Older age (Fig. 5C). These findings suggest that the reorganization of the mitochondria varies along the process of aging, dynamically correlating with jSR-mitochondria interactions. On this regard, we explored the changes in the IP3 receptor type 1 (IP3R1) localization in isolated muscle fibers of mice in the younger and older groups comparing its co-localization with mitochondrial protein Hsp70 (Supplementary Fig. 4A). The results show that the co-localization of these proteins significantly decreased in the Older group when compared with the Juvenile group when evaluated through the Manders coefficient (Supplementary Fig. 4B). These results are consistent with the reduction in SR-mitochondria contacts depicted in Fig. 5.

To further understand if the variations in mitochondrial distribution and topology affect mitochondrial Ca^2+^ handling we measured mitochondrial Ca^2+^ movements in isolated skeletal muscle fibers of the four age-separated groups of mice.

### Mitochondrial Ca^2+^ handling is altered in midlife.

**Figure 6:**
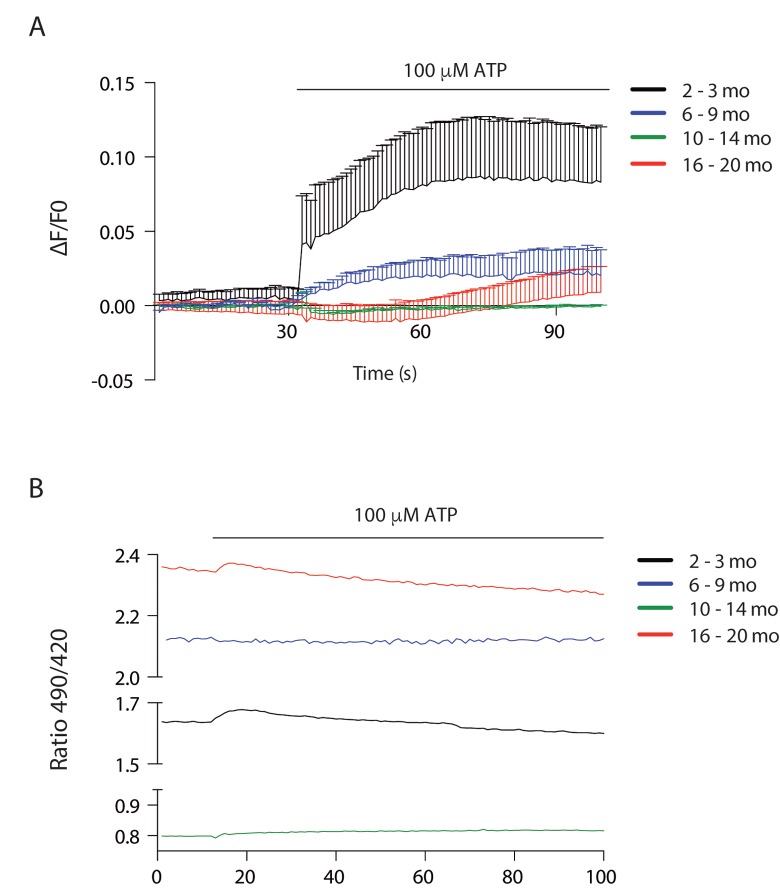
**Cytosolic and mitochondrial Ca^2+^ transients differ according to age** (**A**) Cytosolic Ca^2+^ peak transients are diminished in skeletal muscle fibers from adult and old mice. (**B**) Resting mitochondrial Ca^2+^ levels differ in the different stages of lifespan in mice. Mitopericam data are expressed as the 490/420 nm ratio. Upon ATP stimulation, mitochondrial Ca^2+^ transients were observed. We show representative traces of each age group.

To address Ca^2+^ handling we measured cytosolic signals in isolated muscle fibers of each group after ATP stimulus, we found that a cytosolic signal was obtained only in juvenile mice, which was significantly decreased in the 6-9 mo group. This signal was completely lost in the 2 older groups (Fig. 6A).

To investigate mitochondrial Ca^2+^ changes we used the ratiometric mito-directed plasmid Mitopericam. Our results show that resting mitochondria calcium varies importantly with age (Fig. 6B). Muscle fibers from juvenile mice have an intermediate level that increases about 30% in young adults, correlating with increased muscle function. Resting calcium levels drop dramatically in the 10-14 month old group, again correlating with a drop in muscle function. The older group then shows the highest resting calcium levels (3 fold higher than the previous group) in muscle fibers that have not recovered functional parameters. We also measured the effect of external ATP addition in mitochondria Ca^2+^ (Fig. 6B); a very faint transient increase was evident in the juvenile group, being even smaller in the old group and almost no detectable in the young and older adult groups.

## DISCUSSION

The causes of sarcopenia have been a matter of study for many years and several hypotheses have been put forward; nevertheless, the cellular mechanisms that cause sarcopenia are still a matter of debate. Some authors have defined sarcopenia as the loss of muscle mass and strength without the loss of overall body weight. During the last decades, different groups have focused on muscle size as the primary cause of sarcopenia, but recent findings have demonstrated that size appears not to be a cause and actually plays a minor role [31]. On this regard, sarcopenia can be subdivided into 3 stages: pre-sarcopenia, sarcopenia and severe sarcopenia. Pre-sarcopenia is characterized by a decrease in muscle mass without impact on muscle strength or physical performance. The stage of sarcopenia is characterized by low muscle mass, accompanied by low muscle strength or low physical performance, and finally severe sarcopenia is the stage where the three components: low muscle mass, decreased strength and diminished physical performance can be identified [32].

Based on the aforementioned stages, we investigated how physical capacity and body changes occur over time in four groups of mice of different age, in order to explore how sarcopenia develops before the manifestation of clear muscle systemic symptoms in old mice. Our results show that there is an increase in body weight as the mice develops to an adult stage, together with this there is an increase in adipose tissue that remains over time, whereas muscle weight reaches a peak in the adult stage and then slightly starts to decline. Moreover, strength is significantly decreased only in the older group supporting the notion that sarcopenia is evidenced at late stages of life. However, resistance as a parameter of muscle function is dissociated of muscle mass loss, as it is decreased in the 10-14 months old group, despite muscle mass or strength are not yet decreased. On this regard, our study defines that C57BL/6J mice can effectively be a model for sarcopenia but a precise definition of the stage of each mouse is needed, since the molecular and subcellular mechanisms appear to begin before the symptoms can be evidenced. Supporting these results, it has been described that the weight of skeletal muscles of the hind limbs in rats declines slowly between 6 and 18 months of age, but after that age muscle weight declines rapidly [33].

Moreover, our results support those recently described where 12-month-old mice showed an increase in body weight but a diminished muscle weight/body weight ratio [34]. Altogether, our findings suggest that the onset of changes that may lead to sarcopenia occur earlier than many symptoms and its progression, and clinical manifestations can only be observed when the cellular processes have already started.

In recent years, researchers have been developing a number of novel animal models for the study of cachexia caused by several underlying diseases and also for sarcopenia [35]. Our study focuses mainly on the skeletal muscle, but we cannot rule out other physiological aspects that can contribute to sarcopenia, such as the decrease in oxygen supply in the skeletal muscle that occurs in aging process [36].

On this regard, our model embraces mice from Juvenile to 20 months old mice; in this age range, mice did not present any sign of illness and we can be sure that no pathology is influencing their physical performance or mitochondrial changes; this way the changes reported are likely to be associated to the normal aging process.

Our main finding is that a series of important changes in mitochondria and mitochondria-related proteins occur in parallel with a reduction in resistance exercise performance in 10-14 month old adult mice (see Table 1). At this age, major changes in mitochondria orientation, size and contacts with SR, fission and fusion-related proteins and basal mitochondria calcium content are evident, suggesting that an important adaptive process is taking place. After this age, some of these parameters approach again the values of younger mice, but mitochondria calcium content goes very far up, to values that are likely to cause mitochondria damage if not to trigger pro-apoptotic processes [37].

**Figure:**
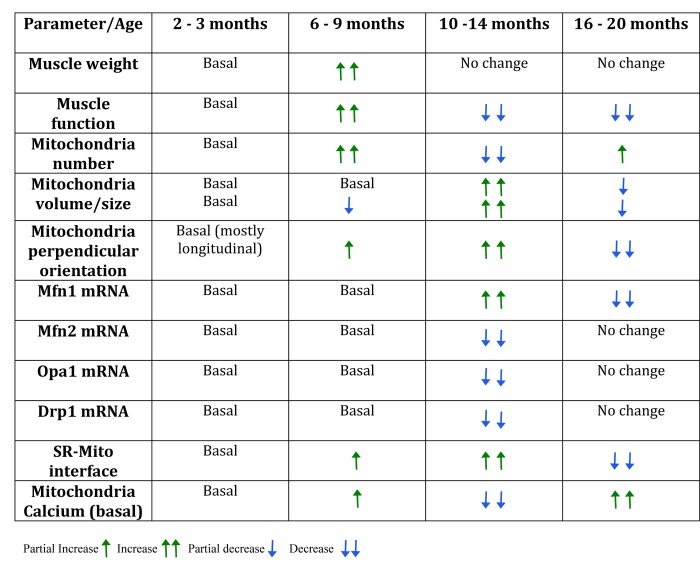
Table 1. Summary table of the changes found in the different groups during mice lifespan.

Previous studies have reported that in skeletal muscle is common to find giant and dysfunctional mitochondria during aging, which are characterized by being highly interconnected and present abnormalities in their ultrastructure [38]. These observations have led to the idea that an imbalance in mitochondrial dynamics occurs during aging in the musculoskeletal tissue. We determined several alterations in mitochondrial topology and distribution during this process being these modifications a scenario previous to a significant loss of muscle mass and force in an old stage.

Mitochondrial morphology has been directly linked with mitochondrial metabolism [39]; however, the study of mitochondrial dynamics and morphology in muscle fibers has been difficult because of the structural characteristics of skeletal muscle cells. Eisner et al. described the occurrence of fusion events in FDB isolated muscle fibers using PA-GFP and mito-ds-red plasmids [22]. Moreover, these events were dependent on Mfn1 and Opa1, with no incidence of Mfn2. Our results show that mRNA levels of Mfn1 but not Mfn2 increase in the 10-14 months group, however the genomic profile performed in rats show a decrease in mRNA levels of all the proteins involved directly in mitochondrial morphology starting at 12 months old [33]. On this regard, our results show that Opa1 levels are decreased in the older groups (10-14 and 16-20); this could be a cause of incomplete fusion or incompetent mitochondrial cristae. These two phenomena have been directly linked with impaired mitochondrial function, described as one of the possible causes of aging and lately being a matter of debate in relation with muscle atrophy [40]. Moreover, mRNA levels of Drp1, involved in the fission process are significantly decreased in the 10-14 months group, suggesting that the balance between fission and fusion proteins may be altered during this period of life.

Skeletal muscle contains two different mitochondrial subpopulations: the SSM, which are located directly below the sarcolemma, and IMF, arranged in parallel rows between the myofibrils [41]. Both subpopulations of mitochondria communicate, possibly to provide a conductive pathway of energy distribution among skeletal muscle fibers [25]. Our results show that mitochondrial morphology changes during the aging process and those different stages can be found. As there is growth between 2-3 months and 6-9 months old, the increased number of mitochondria is probably due to an increase in biogenesis to enhance metabolism. After the growth process and together with the peak in body weight, we found an increase in the volume of mitochondria accompanied by a decrease in the number of elements, which can be considered a fused-like phenotype. On this regard and supporting our results, Sayed et al. identified larger mitochondria by EM in Gastrocnemius muscle of 12-month-old mice [34]. After this stage, the oldest group of 16-20 months presented an increase in the number of mitochondria but also a decrease in the volume of each object, supporting the idea of a fissioned phenotype, probably associated to mitochondrial dysfunction. Similar results in older than 24 months old mice have been described [42]. This suggests an imbalance in the process of fusion/fission favoring early mitochondrial fusion during aging in skeletal muscle [20]. Some research has demonstrated that the use of a transgenic mouse model with overexpression of the fission machinery was sufficient to induce mitochondrial dysfunction, remodeling of the mitochondrial network, protein degradation and atrophy of the muscle fiber [43]. This led to propose that excessive activation of mitochondrial fission processes might induce mitochondrial dysfunction and subsequent muscle atrophy, under certain experimental paradigms [44]. On the other hand, some studies have reported that promoting mitochondrial fission in midlife can prolong lifespan in a model of *Drosophila melanogaster.* On this regard, these results support the hypothesis that at the age 10-14 months (midlife) there is a major change of the mitochondrial network that precedes mitochondrial dysfunction and muscle decline [45]. Moreover, in a model of *C. elegans* inhibiting mitochondrial dynamics blocks AMPK- and dietary restriction-mediated longevity [46].

Other studies have established that mitochondrial morphology depends on muscle fiber type, on this case Mishra et al. described that fusion rates are determined by fiber type and can vary with exercise and aging. Moreover, Picard et al. determined that mitochondrial function is not causally related with muscle atrophy, but it can be related to muscle fiber type [21]. Our study focuses on a model of FDB muscle, a mainly type II fiber muscle, these fibers appear to be the first to change during the aging process [47].

We also described that both the distribution and orientation of mitochondria changed with age, the most prominent change occurring at 10-14 month old when there is redistribution towards a more striated pattern, perpendicular to the longitudinal axis of the fiber. After this period, more longitudinal mitochondria can be found: these results concur with those in fibers from aged mice where, striations are not as well preserved as in adult fibers, with some mitochondria forming longitudinal columns between myofibrils [42].

The tight link between Ca^2+^ and excitation-contraction (EC)-coupling has also been focus of research during the aging process and particularly in already old models. Impaired EC coupling function has been described in aged muscle resulting in a reduced supply of Ca^2+^ ions to the contractile elements, and thus, reduced specific force [48]. These findings correlate with previous studies referring to impaired Ca^2+^ release in aged muscle and reduced SR Ca^2+^ release in SR vesicles [49]. In addition, other defects such as uncoupling between the voltage sensor Cav1.1 and RyR1 may also contribute to muscle weakness in aging [50]. Age-dependent uncoupling of mitochondria from the Ca^2+^ release units was proposed [42]. They described an increase in damaged mitochondria together with reorganization towards the SR, with a significant decrease in tethers, which were misplaced from their normal triad position, possibly resulting in reduced metabolic efficiency and a consequent decline in skeletal muscle performance.

We have demonstrated alterations in mitochondrial Ca^2+^ handling as a function of age and we see an increase in mitochondrial fusion and size, repositioning of mitochondria to maximize contact with SR and complete buffering of IP3-dependent cytosolic Ca^2+^ by mitochondria in adults. Together with this adaptive process, mitochondria resting Ca^2+^ levels dramatically drops in the Adult group compared to the Young and the juvenile muscle; whether these changes in resting Ca^2+^ are related to the morphological changes seen at this age, needs to be further studied. After 16 months of age, this process appears to recede and we found again a fission-like phenotype, decreased mitochondrial number and size, reduced jSR-to-mitochondria pairs and elevated resting mitochondrial Ca^2+^ levels. Ca^2+^ transfer between the SR and mitochondria has been characterized and independent signals in the different compartments involved in the process have been measured; a very small cytosolic signal can be present in parallel with a large mitochondria transient [51]. It is tempting to conclude that these later changes lead to a reduced mitochondria function in aging. It has been recently described that MCU, the master regulator of mitochondrial Ca^2+^ uptake is sensitive to exercise in aging humans [52], suggesting that these signals are indeed important for muscle function and adaptive processes during aging.

More research will be needed to firmly establish the relationship between mitochondrial dynamics, mitochondria morphology and orientation as well as mitochondrial Ca^2+^ handling during the aging process to pinpoint the consecutive appearance of sarcopenia symptoms; for the moment, increasing evidence point to important changes in mitochondria that will necessarily alter metabolic regulation and efficiency along the aging process.

We have described here the timeline modifications of the mitochondrial morphology during the process of aging in skeletal muscle. We demonstrated that non-linear changes in fusion and fission-like phenotypes as well as in orientation of mitochondria correlate with changes in the SR-mitochondria communication and in mitochondria Ca^2+^ handling. These changes appear also to correlate with changes in muscle function that are evidenced at the different stages of mice life. These findings, strongly suggest the idea that mitochondrial topology and morphology are involved in the process of sarcopenia and loss of muscle function during aging, a process that appears to start well before old age. Moreover, we established that major changes occur in a stage of life before those changes that can be considered actual symptoms of sarcopenia, which brings up the necessity of further studying skeletal muscle physiology at different ages and upon early interventions in order to delay the onset of sarcopenia.

## MATERIALS AND METHODS

### Animals

Male C57BL/6J mice were housed in temperature-controlled rooms (21°C), on a 12-h light/dark cycle. This study was carried out in accordance to the ethics committee of the Faculty of Medicine, University of Chile.

### Inverted grip test

Muscle strength of mouse limbs was tested with an inverted grip-hanging test [53]. Mice were placed individually on the center of a 25x25 cm wire grid (wire width <0.1 cm and spacing 0.5 cm), mounted 35 cm above a soft platform. After gently inverting the grid, the mouse hanging time was recorded (grip latency). This procedure was repeated three fold and the average hanging time values were calculated for each mouse. Data is presented in terms of impulse, which implies the normalization of each mouse to its weight in order to compare the force developed in each assay.

### Treadmill exercise

Animals were first conditioned in the treadmill with a protocol of 0.5 km/h for 5 minutes for three consecutive days before submitting them to exercise. We used an incremental speed protocol that started at a speed of 0.5 km/h for 3 minutes and then speed was increased by 0.2 km/h every 2 minutes until exhaustion to calculate the maximal speed reached. After a day of rest, the resistance test was performed. We used a protocol that started at a speed of 0.5 km/h for 3 minutes and then speed was increased to 80% of the maximum speed previously reached by each mouse. The test was performed until exhaustion and time was recorded.

### FDB muscle electroporation

Intramuscular plasmid injection and electroporation were performed according to Schertzer et al*.* [54]. In brief, the animals were anesthetized using 5% isoflurane to inject their footpads with 2 mg/ml hyaluronidase type IV (Sigma-Aldrich Co.) and injected 1 h later with 20 μg Mito-ds-red plasmid (Takara Bio Inc, USA). Electroporation was then performed using acupuncture needles as electrodes and delivering 20 pulses of 100 V and 20-ms duration at 1 Hz using a pulse stimulator (Grass S48; W. Warwick, RI, USA).

### Isolation of adult skeletal muscle fibers

Isolated fibers from the *flexor digitorum brevis* (FDB) muscle were obtained by enzymatic digestion with collagenase type II (90 min with 400 U/ml; Worthington Biochemicals Corp., Lakewood, NJ, USA), and mechanic dissociation with fire- polished Pasteur pipettes, as described previously [55]. Isolated fibers were seeded in Matrigel-coated dishes and used 20 h after seeding.

### Histology

Serial cryosections (12 μm thick) from adult mice muscle were fixed using freshly prepared paraformaldehyde (4%) for 30 min and washed in distilled water. Subsequently, Hematoxylin / Eosin techniques were performed to evaluate the general state of muscle tissue. We performed a Van Gieson’s trichromic technique to evaluate the state of fibrosis. Histological preparations were examined by bright field microscope (Leica microsystems DM 500 – camera ICC50W) and 5 images were obtained of each preparation. These ones were later analyzed with image J (NIH).

### Confocal imaging

Isolated muscle fibers expressing Mito-ds-red were incubated in Krebs solution. Confocal image stacks (average 45 z-slices, 1 μm) were captured with a Nikon C2 confocal microscope, using a 60X Plan-Apochromatic λ CFI oil (1, 4) objective. Each experiment was repeated in at least three mice for each group, and 16–25 cells per condition were quantified in each experiment.

### Mitochondrial morphology analysis

Z-stacks were deconvoluted, thresholded, and 3D-reconstructed using ImageJ software (NIH). Number and volume of individual mitochondria were quantified using the 3D Object Counter plug-in as previously described by Parra et al. 2014 [56]. A theoretical model of the Landweber deconvolution algorithm and the PSF (Point Spread Function) corresponding to the experimental conditions were applied to the stacks obtained with the mt-DsRed plasmid. In detail, the images were manually fixed with a threshold, which was used to segment the image between voxels corresponding to the mitochondrial network and voxels corresponding to background noise, each threshold was done in blind and by two different people. The number of mitochondrial voxels (pixels^3^) multiplied by the volume of each voxel results in total mitochondrial volume. Subsequently, the algorithm groups the voxels that are contiguous with each other. Each group of contiguous voxels is recognized as an individual mitochondrion. In this way, the number of groups corresponds to the number of mitochondria in the cell, and the total volume divided by the number of mitochondria corresponds to the average size of the mitochondria (Parra et al., 2008).

### Image segmentation

A semi-automatic segmentation was applied to the muscular cell images. First a Gabor filter bank [57] using 6 orientations (θ=0°, 30°, 60°, 90°, 120°, 150°), 6 scales (σ_x_=0.1, 1.1, 2.1, 3.1, 4.1, 5.1; σ_y_= σ_x_/gamma), and parameters gamma=0.5, and lambda=4 σ_x_ was applied to the set of images, obtaining 36 filtered images. At each pixel location the maximum intensity of all 36 images was selected. Over the maximum intensity image a manual threshold chosen by the biologist expert was applied for fibers segmentation. Using the information of which of the 36 images was the maximum value associated to each pixel was associated to one of the 6 orientations.

### Parameter computation

The distribution of the orientation (with respect to the fiber major axis) of each of the fibers pixels was estimated. Relative orientation was obtained by first, segmenting the complete cell to obtain the cell main axis [58] and then subtracting cell orientation to each pixel orientation. To compare pixel-fibers orientation distribution of multiple cells histograms were divided into 3 or 8 bins, where 0 represents the total number of pixels associated to fibers in the same orientation as the cell. The number of bins was selected such as to avoid empty bins. Each bin is represented as a different color in each image shown in the right panel of Fig. 2A.

### Cytosolic Ca^2+^ measurements

Cytosolic Ca^2+^ was measured using Fluo-4 AM probe. In brief, isolated skeletal muscle fibers were seeded in 25 mm coverslips and incubated for 20 minutes with Fluo-4 AM (5 µM) afterwards, fibers were washed with Krebs solution and placed in a microscope-fit chamber. Spinning disk microscopy in time-lapse mode was used to capture 30 s of changes in fluorescence after a 100 μM ATP stimulus.

### Mitochondrial Ca^2+^ measurements

FDB muscles were electroporated with Calcium sensitive mitochondrial matrix-targeted Mitopericam construct. Images of isolated muscle fibers were collected for 560 seconds using Olympus® IX81 Spinning disk microscope in a time-lapse mode (acquisition frequency 2s), ex. 490 and 420 nm, em. 510 nm. Prior to stimulation, the fibers expressing Mitopericam were incubated at 37 ° C for 30 min in Krebs Ringer buffer. ATP (100 μM) was added to the microscopy chamber as indicated in Fig. 6. Data were analyzed using ImageJ software. We corrected the baseline for every sequence of images by subtraction of a pre-stimulus fluorescent image. The mean intensity of 3–6 regions of interest per cell, located in the mitochondria, were collected to represent fluorescence changes with time (F(t)). Signals are presented as the ratio of 490/420 nm of Mitopericam. 4-5 independent fibers were registered of at least 3 different mice per group. Data is shown as a representative curve for each experiment.

### Quantitative real time RT-PCR assay

Total RNA was obtained from skeletal muscle fibers employing Trizol reagent (Ambion, Life Technologies, USA) according to manufacturer´s protocol. The concentration and purity of RNA were determined by absorbance at 260/280 nm. 1 μg of total RNA was reverse transcribed using a high-capacity cDNA reverse transcription kit (Invitrogen, Thermo Fischer Scientific, USA) according to the manufacturer’s instructions.

Real-time PCR was performed using Stratagene Mx3000P (Stratagene, La Jolla, CA, USA) using Brilliant III Ultra-Fast SYBR QPCR master mix amplification kit (Agilent Technologies, Santa Clara, CA, USA). The primers used were: Mfn-1 5’-ATTGGGGAGGTGCTGTCTC-3’ (sense), 5’-TTCGGTCATAAGGTAGGCTTT-3’ (antisense); Mfn-2 5’-ATGTTACCACGGAGCTGGAC-3’ (sense), 5’-ACCTGCTTCTCCGTCTGCAT-3’ (antisense); Opa-1 5’-GATGACACGCTCTCCAGTGAAG-3’ (sense), 5’-CTCGGGGCTAACAGTACAACC-3’ (antisense); Drp-1 5’-GTTCCACGCCAACAGAATAC-3’ (sense), 5’-CCTAACCCCCTGAATGAAGT-3’ (antisense); Fis-1 5’-AAGTATGTGCGAGGGCTGT-3’ (sense), 5’-TGCCTACCAGTCCATCTTTC-3’ (antisense); b-actin 5’-TCTACAATGAGCTGCGTGTG-3’ (sense); 5’-TACATGGCTGGGGTGTTGAA-3’ (antisense).

A typical reaction contained 250 nmol/l of forward and reverse primer, 1 μl cDNA and the final reaction volume was 20 μl. All primers used presented optimal amplification efficiency (between 90% and 110%). PCR amplification of the housekeeping gene b-actin was performed as a control. Thermocycling conditions were as follow: 95˚C for 5 min and 40 cycles of 90°C for 15 s, 60°C for 15 s, 72°C For 15 s. Expression values were normalized to b-actin and are reported in units of 2^−ΔΔC^ + s.d. as described (Pfaffl, 2001). CT value was determined by MXPro software when fluorescence was 25% higher than background. PCR products were verified by melting-curve analysis.

### Western blotting

Tissue samples were homogenized in a lysis buffer containing 20mM Tris-HCl (pH 7,5), 1% Triton X-100, 1mM EDTA, 1mM EGTA, 20mM NaF, 1mM Na2P2O7, 10% glycerol, 140mM NaCl, 10mM Na3VO4, 1mM PMSF and protease inhibitors (Complete Mini, Merck, USA). Tissue lysates were centrifuged at 12,000 rpm for 10 minutes at 4°C to remove insoluble material. Protein concentration was determined by Bicinchoninic Acid (BCA) Assay (Thermo Scientific, USA). 20ug proteins were separated by sodium dodecylsulphate- polyacrylamide gel electrophoresis (SDS-PAGE) on 12% resolving gels and electro transferred to nitrocellulose. Membranes were blocked with 5% milk-TBS-T. Membranes were incubated with primary antibodies at 4°C and blotted with horseradish peroxidase-linked secondary antibodies (1:5,000 in 1% (wt/vol) milk in TBS-T). Signals were detected using ECL Supernova kit (Cyanagen, Bologna, Italy) and quantified by scanning densitometry (Chemidoc Biorad, USA). Protein content was normalized with Ponceau red.

### Transmission electron microscopy

Skeletal muscle samples were prepared as described previously [22] with few modifications. In brief, mice were killed via decapitation and whole feet without skin were fixed in 2.5% glutaraldehyde for 2 h at room temperature and overnight at 4°C. The next day, FDB muscle was dissected into small bundles of fibers, washed four times with 0.1 M sodium cacodylate buffer, and stained with 2% osmium tetroxide in 0.1 M sodium cacodylate buffer for 2 h. Then the samples were washed with water and stained with 1% uranyl acetate for 2 h. Stained samples were dehydrated on an acetone dilution series and embedded in epon. 80-nm thin sections were cut from the embedded specimens, mounted on electron microscopy grids, and examined using a transmission electron microscope Philips, Tecnai 12 at 80 kV. Morphometric analysis of IMF was performed on longitudinal sections using Fiji/ImageJ software. Quantification of the number, size and density of mitochondria were measured in 8.200×. The number of mitochondria-to-SR pairs and length of SR-mito interface were measured in 20,500×. The length of jSR-mito interface was determined as the % of mitochondrial perimeter that faces jSR cisternae when the gap among organelles is ≤50 nm [59].

### Statistical analysis

Data of n cells (n ≥ 5) obtained from at least 3 independent experiments were expressed as mean ± SE and analyzed by one-way ANOVA. p value < 0.05 was considered statistically significant (IC 95%). All statistical analyses were performed using GraphPad Prism 5.

## Supplementary Material

Supplementary File
