# Supplementary material for: Muscle function decline and mitochondria changes in middle age precede sarcopenia in mice
Source: Aging (Albany NY). 2018 Jan 4;10(1):34–55. doi: 10.18632/aging.101358 (PMC5811241; doi:10.18632/aging.101358)
Supplement: Supplementary File [file aging-10-101358-s001.pdf]

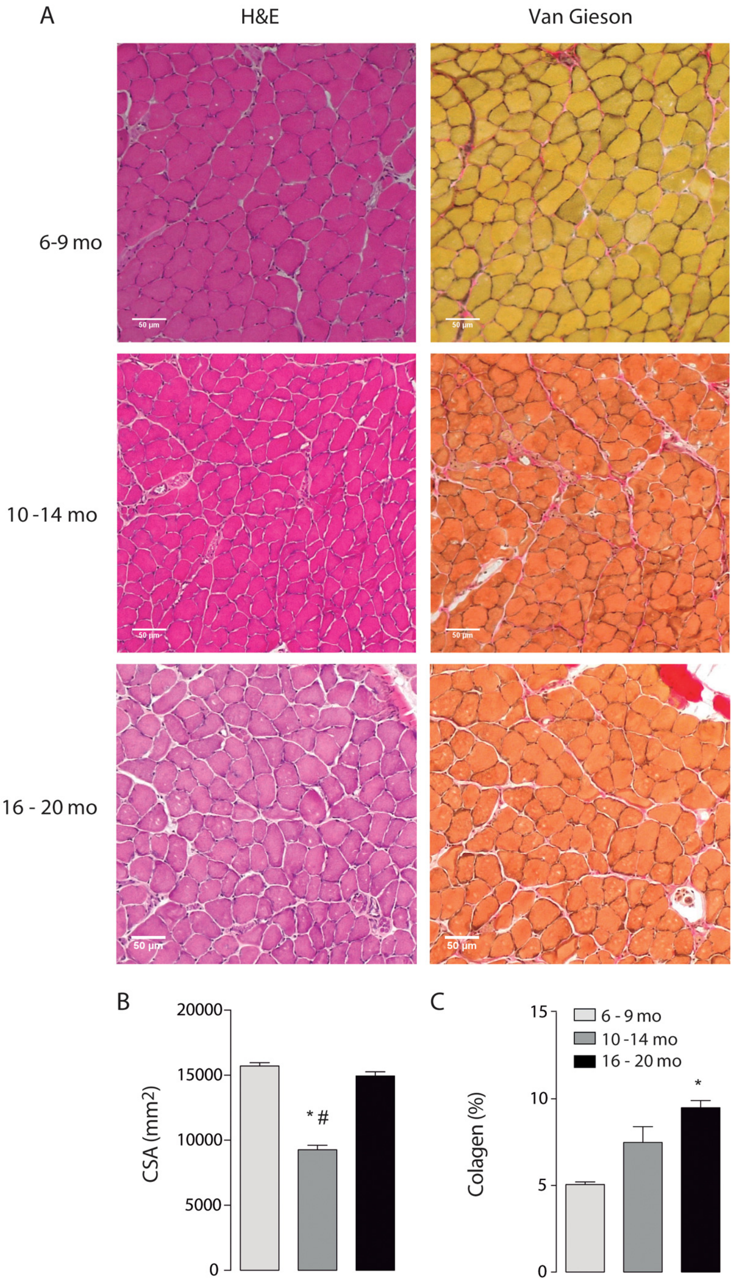

Supplementary Figure 1. Changes in cross sections of skeletal muscle appear in midlife mice. (**A**) Representative images of cross sections of Gastrocnemius/soleus complex in the different groups of mice (**B**) Cross-sectional area determined by Hematoxilin/eosin in the Gastrocnemius/soleus complex is significantly decreased in 10-14 mo group. (**C**) Collagen was determined by Van Gieson and is significantly increased in 16-20 mo group.

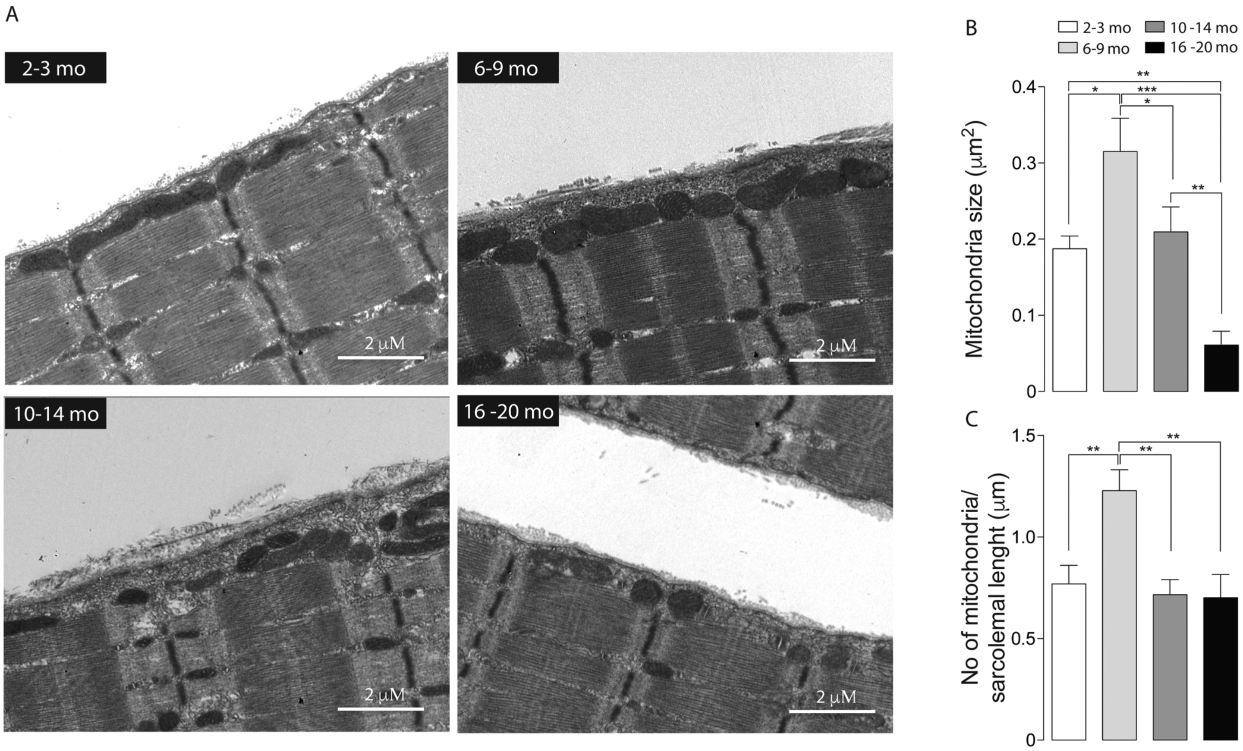

Supplementary Figure 2. Decreased subsarcolemmal mitochondrial size in aged mice. Each bar represents the mean ± SD.*p<0.05. **p<0.01 ***p<0.001 (**A**) Representative images of electron microscopy from FDB longitudinal slices from mice from the different groups (**B**) Mitochondria size is significantly diminished in older groups. (**C**) Number of mitochondria per sarcolemma length is significantly increased in the adult group.

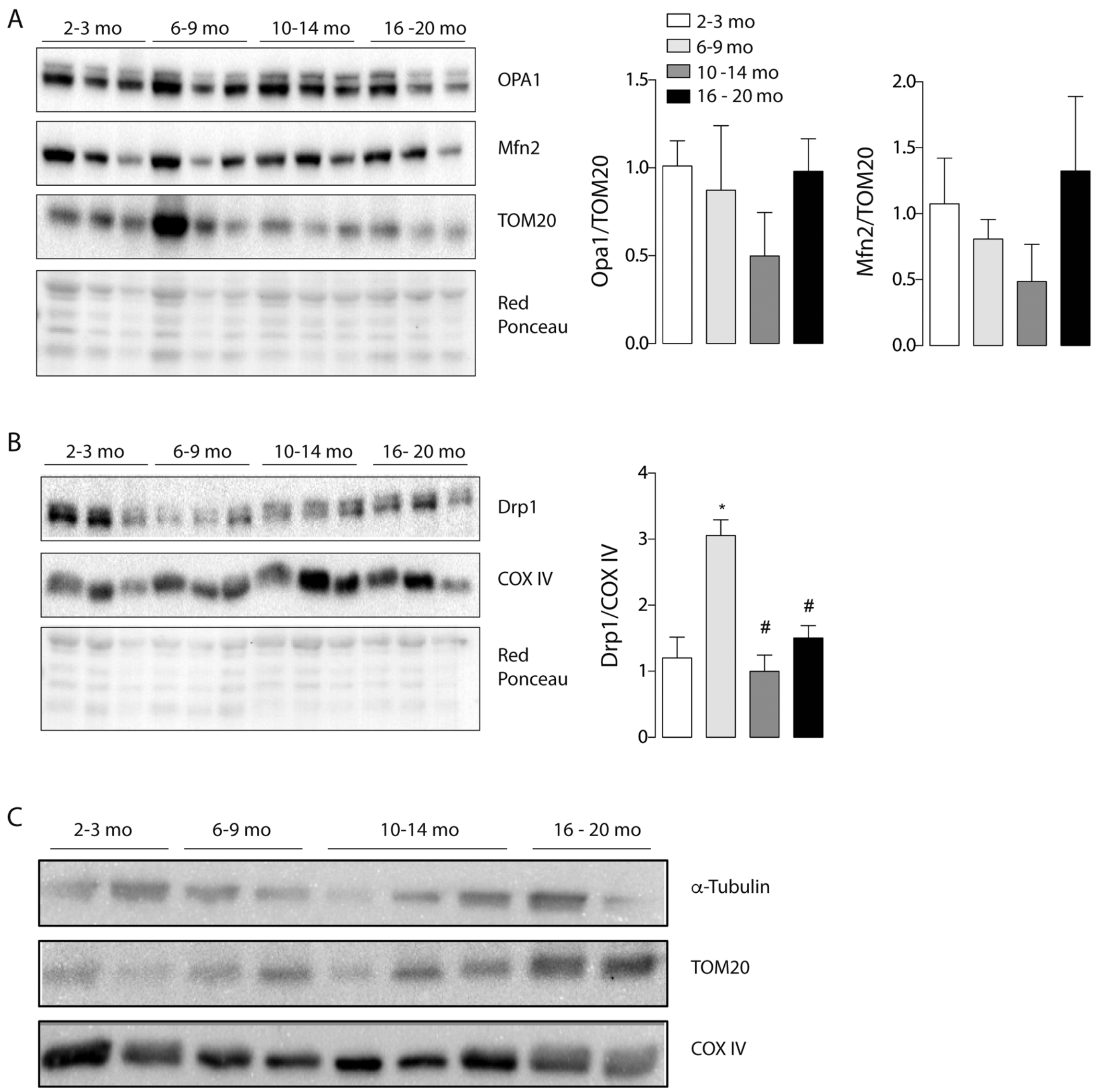

Supplementary Figure 3. Mitochondrial proteins levels are not significantly different in aged mice. (**A** - **B**) Western Blot for mitochondrial proteins in FDB muscle of mice in different groups of age do not vary significantly when normalized by non-related mitochondrial dynamics proteins. (**C**) Western Blot for TOM20 and COX IV in Soleus muscle.

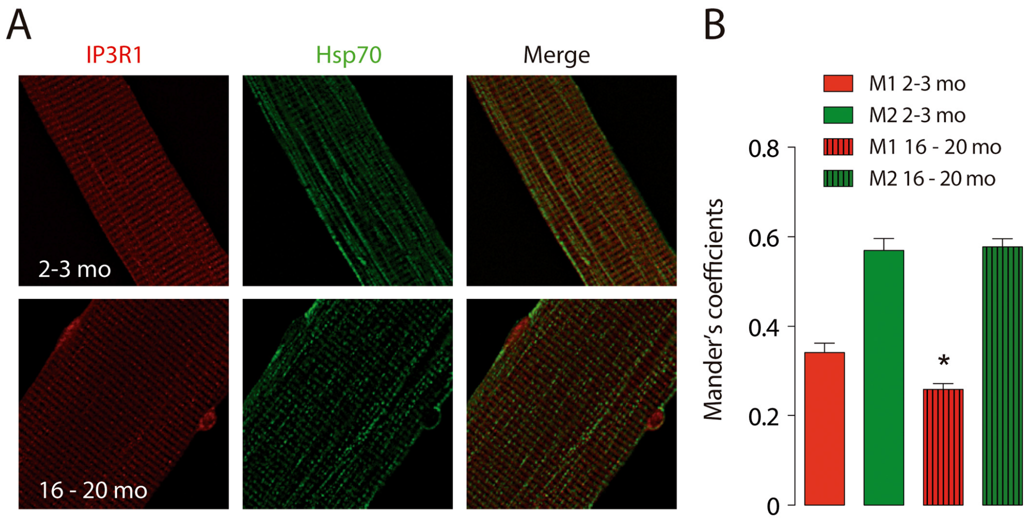

Supplementary Figure 4. Co-localization of IP3R1 and Hsp70mt decreased in aged mice. (**A**) Representative images of muscle fiber inmunofluorescence towards IP3R1 (red), Hsp70mt (Green) and merge (right panel) of Juvenile (2-3 months) and Old (16-20 months) mice. (**B**) Mander´s coefficient quantification decreased in old mice. (Fibers were fixed, permeabilized, blocked, and subsequently incubated with primary antibodies (anti-mtHsp70 or IP3R1). Images were taken by confocal microscopy in a Zeiss LSM-5, Pascal 5 Axiovert 200 microscope. For the co-localization analysis one focal plane was analyzed.
